# Automated fastener versus manually tied knots in minimally invasive mitral valve repair: impact on operation time and short- term results

**DOI:** 10.1186/s13019-015-0344-4

**Published:** 2015-11-03

**Authors:** Martin T. R. Grapow, Miroslawa Mytsyk, Jens Fassl, Patrick Etter, Peter Matt, Friedrich S. Eckstein, Oliver T. Reuthebuch

**Affiliations:** 1 Department of Cardiac Surgery, University Hospital Basel, Spitalstrasse 21, CH-4031, Basel, Switzerland; 2 Department of Anesthesia, University Hospital Basel, Spitalstrasse 21, CH-4031, Basel, Switzerland

**Keywords:** Cor-Knot, Automated fastener, Minimally invasive mitral valve repair, Manually tied knots, Cross-clamp

## Abstract

**Background:**

This study compares the influence of two different annuloplasty attachment suture applications, namely the use of an automated fastener versus manually tied knots using a traditional knot pusher, on total operation time, on cardiopulmonary-bypass time and on cross-clamp time, and on short-term outcome.

**Methods:**

Sixty patients underwent isolated minimally invasive mitral valve repair in Carpentier Type-II mitral disease with implantation of an annuloplasty ring in combination with correction of the prolapsing leaflet using artificial chords. The first 30 patients after implementation of a novel automated fastener were compared with the last 30 patients corrected with a traditional knot pusher. No significant differences with regard to demographic data (age, gender, NYHA class, ejection fraction, BMI, cardiovascular risk factors) between the two groups were found. All patients received isolated mitral valve repair in the first run. Bretschneider HTK was used for cardioplegic cardiac arrest in all patients.

**Results:**

Transesophageal and transthoracic echocardiography at the end of operation and at discharge revealed no (*n* = 25), trace (*n* = 28) or mild (*n* = 7) residual regurgitation with no evidence of ring dehiscence and without any significant clinical differences between the groups. Cross-clamp, cardiopulmonary-bypass and total- operation time were significantly reduced in the automated fastener group compared to the group using a traditional knot pusher (87.1 ± 17.9 vs. 101.3 ± 17.8; *p* < 0.01, 138.1 ± 25.6 vs. 152.7 ± 24.9; *p* < 0.05, and 203.9 ± 31.02 vs. 223.8 ± 29.01; *p* < 0.01, respectively).

**Conclusion:**

Our short-term results indicate a safe, reliable and fast application of the novel automated fastener device in combination with significant time savings in cardioplegic arrest and cardiopulmonary bypass.

## Background

Mitral valve dysfunction is the second-most common clinically significant form of valvular defect in adults [1]. During the last 15 years, minimally invasive mitral valve surgery (MIMVS) using the right anterolateral mini-thoracotomy access has emerged as an accepted approach for the management of mitral valve disease alone or in combination with tricuspid valve disease, defects of the atrial septum or atrial rhythm disorders with equivalent results [2]. This strategy was developed to decrease surgical trauma by minimizing the size of incisions. It permits excellent exposure of the mitral valve, thereby avoiding conventional full sternotomy. This results in better visualization of the valve for the surgeon and even for all other participants in the OR due to transmission via monitor, faster mobilization of the patient, faster healing, less pain, less infection and better cosmetics. One constraint of minimally invasive surgery is the need for remote knot tying, which is typically accomplished with the use of a knot pusher [3]. The challenge of video-assisted knot tying, at disadvantageous angles, has long been recognized as one important drawback in the advancement of minimally invasive surgery. The main problem is that knot tying using endoscopic or robotic-assisted tools is time-consuming, leading to increased operation times [4, 5]. Operating time is especially critical for cardiac procedures involving cardiopulmonary bypass (CPB). Increased CPB times lead to higher risks of multiple organ dysfunction syndrome [6], and therefore adverse outcomes [7]. Patient mortality rates increase with the cross-clamp time, especially in older patients and during emergency procedures. Furthermore, older patients and otherwise “unhealthy” patients represent the majority of candidates requiring surgical procedures. The Cor- Knot automated fastener (LSI SOLUTIONS, Victor, NY, USA) was introduced in our hospital in November 2013. The aim of our retrospective study is to investigate the influence of manual and automated knot-tying systems on cross-clamp, CPB and total operation time in one defined operative procedure: isolated minimally invasive mitral valve repair via a right anterolateral, endoscopically assisted thoracotomy with femoral cannulation for the cardiopulmonary bypass (CPB) in Carpentier Type-II pathology.

## Methods

### Patients

We started our minimally invasive mitral valve program at University Hospital Basel in 2010, reaching a volume of 60 – 70 patients per year. From a total of 69 patients who received mitral valve repair in a minimally invasive setting from May 2013 until June 2014, 60 patients we evaluated retrospectively. These patients underwent isolated minimally invasive mitral valve repair in Carpentier Type-II mitral disease, all of them having an annuloplasty ring implanted in combination with correction of the prolapsing leaflet using artificial chords (GoreTex CV4). Repair was performed in the first run without the need for any further correction. Thirty patients after implementation of a novel automated fastener (November 2013– June 2014) were compared with 30 patients corrected with a traditional knot pusher (May 2013– October 2013). To increase homogeneity in both groups the following 9 patients were excluded: Patients, who underwent combination surgery with tricuspid valve repair or atrial septal defect closure (*n* = 3), Patients just receiving a ring- annuloplasty (*n* = 5) and one patient who needed a second cross-clamp period for a suboptimal result in the first run. No significant differences regarding demographic data (age, gender, NYHA class, ejection fraction, BMI, cardiovascular risk factors) between the two groups were found (Table 1). Three patients presented with an anterior leaflet prolapse in the knot-pusher group compared to 4 patients in the Cor-knot group. All other patients had the prolapsing segment located at the posterior leaflet. The study was approved by our local ethics comity.

**Table 1:** Patients’ characteristics

Characteristic	Manual	Cor-Knot	*p*
No. of patients	30	30	n.s.
Age	56.13 ± 16.11	58.8 ± 13.84	n.s.
Male	24 (80 %)	17 (57 %)	n.s.
Female	6 (20 %)	13 (43 %)	n.s.
BMI	25.96 ± 3.58	24.95 ± 6.38	n.s.
Height	176 ± 9.1	174 ± 10.3	n.s.
Weight	80.5 ± 12.1	76.5 ± 20.4	n.s.
Diabetes	1 (3.3 %)	1 (3.3 %)	n.s.
Smokers	4 (13.3 %)	2 (6.6 %)	n.s.
Dyslipidemia	6 (19.9 %)	9 (29.7 %)	n.s.
Hypertension	15 (49.5 %)	22 (72.6 %)	n.s.
Anterior leaflet prolapse	3 (10 %)	22 (72.6 %)	n.s.
Anterior leaflet prolapse	3 (10 %)	4 (13.3 %)	n.s.
Ejection fraction	60.73 ± 9.20	58.8 ± 8.63	n.s.
Euroscore 2	1.03 ± 0.64	1.21 ± 0.85	n.s.

### Operative technique

Minimally invasive mitral valve surgery is performed through a right mini-thoracotomy using standardized methods. Briefly, standard monitoring lines are inserted and the patient is intubated with a double-lumen endotracheal tube. Access to the thoracic cavity is achieved via a 4–6 cm incision in the inframammary groove that is carried out through the third or fourth intercostal space. A soft tissue retractor is used to limit rib spreading. CPB is set up routinely with femoral vessel cannulation. The opened pericardium is retracted toward the right. Cardiopulmonary bypass is initiated with intrathoracic clamping of the aorta (Glauber-Aortic-Clamp), and the heart is arrested with antegrade infusion of crystalloid cardioplegia (Bretschneider Solution, Custodiol^®^). After cardiac arrest, the left atrium is entered along the interatrial groove. Mitral valve repair is performed using standardized techniques (Annuloplasty ring, Neo Chordae, Gore-Tex Loops). Carbon dioxide field insufflation and standardized transesophageal echocardiography-guided de-airing techniques are routinely used in our clinic. After atrial closure and weaning from CPB, a complete postoperative transesophageal echocardiographic study is recorded. Post-repair echocardiographic data are obtained after closure of the incision.

### The automated fastener

**Fig. 1:**
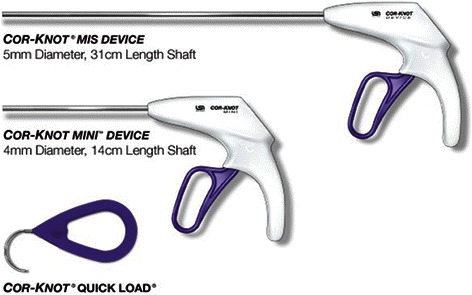
The Cor-Knot.Device

The Cor-Knot device is indicated for use in the approximation of soft tissue and prosthetic materials, used in conjunction with either specified 2–0 polyester or 2–0 polypropylene or 3–0 polypropylene suture and a Cor-Knot quick load. The pusher can be released only under tension (Fig. 1). The fastener is made of titanium.

### Statistical analysis

Continuous data are presented as mean with standard deviation (SD). Categorical data are presented as absolute numbers and percentages. Differences between groups are calculated with the Student’s *t*-test. Overall significance is attained with a *p* < 0.05. The analysis was performed with the use of Microsoft Office Excel (Microsoft, Redmond, WA, USA).

## Results

Transesophageal and transthoracic echocardiography at the end of operation and at discharge revealed no (*n* = 25), trace (*n* = 28) or mild (*n* = 7) residual regurgitation with no evidence of ring dehiscence and without any significant differences between the two groups. Furthermore, no differences were detected in ICU stay, total length of hospital stay or the need for blood transfusion (Table 2). There were no myocardial infarction, stroke, limb ischemia or respiratory problems noted in these patients. All patients survived the procedure and were discharged from hospital.

**Table 2:** Outcome

Characteristic	Manual	Cor-Knot	*p*
Intensive care Unit (days)	2.5 ± 2.33	2.2 ± 1.65	n.s.
Hospitalization duration (days)	10.0 ± 5.42	11.6 ± 12.2	n.s
Blood products per patient	0.7 ± 1.68	0.55 ± 1.12	n.s.

**Fig. 2:**
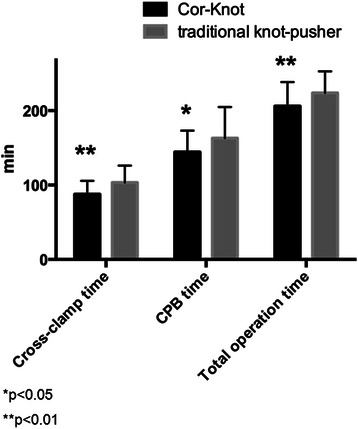
Operative target times

**Fig. 3:**
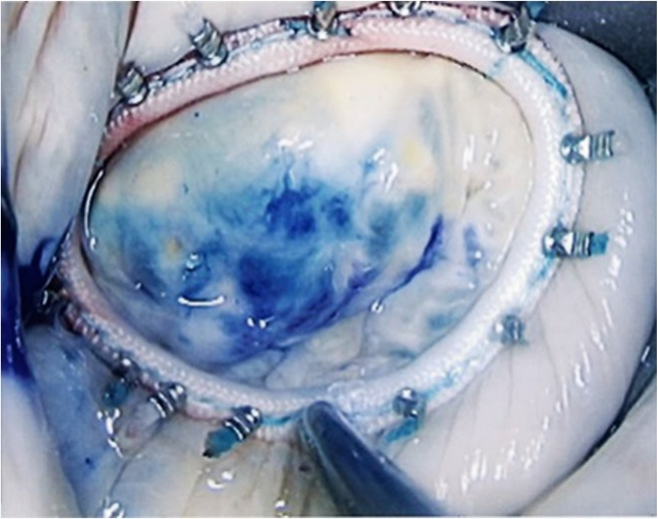
Mitral valve after repair and ring annuloplasty with Cor-Knot fixation

**Fig. 4:**
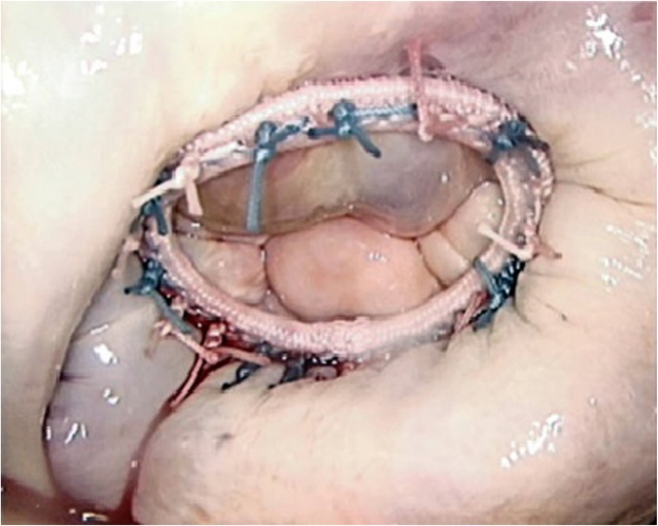
Mitral valve after repair with traditional ring fixation using a knot pusher

The amount of sutures needed for ring fixation were similar in both groups, either in total or as analyzed per patient. Cross-clamp time, CPB time and operation time were significantly reduced in the automated fastener group compared to the group using a traditional knot pusher (87.1 ± 17.9 vs. 101.3 ± 17.8; *p* < 0.01, 138.1 ± 25.6 vs. 152.7 ± 24.9; *p* < 0.05, and 203.9 ± 31.02 vs. 223.8 ± 29.01; *p* < 0.01, respectively). (Table 3, Figs. 2, 3 and 4.)

**Table 3:** Intraoperative data

Characteristic	Cor-Knot	Manual	*p*
No. of knots, total	455	442	n.s.
No. of knots, per patient	15.1 ± 1.86	14.7 ± 1.9	n.s.
Cross-clamp time	87.1 ± 17.9	101.3 ± 17.8	<0.01
ECC time	138.1 ± 25.6	152.7 ± 24.9	<0.05
Operation time	203.9 ± 31.02	223.8 ± 29.01	<0.01

## Discussion

The apparent advantages of minimally invasive mitral surgery through right mini- thoracotomy are considered to be a very small wound surface area, rapid recovery with shorter ventilation time, shorter hospital stay and less blood transfusion, as well as reduced postoperative pain, and better cosmetic result [8]. Furthermore, perfect visualization of the valve in high definition and live transmission via multiple monitors guarantee access for all participants in the OR. The valve site is not only seen by one single surgeon; in fact, more eyes can follow the operation with a possible impact on safety – even teaching is much more feasible. The drawbacks of this technique are perfusion-related complications (e.g. stroke, vascular damage and limb ischemia), which tend to occur more frequently in minimally invasive technique than with the standard technique [9]. In addition, valve repair through a small thoracotomy is technically demanding, leading to prolonged cross-clamp, CPB and operation times with a potential negative impact on outcome.

The results of our study show that with the introduction of the Cor-Knot device (Fig. 3 in our hospital (November 2013), cross-clamp time, CPB time and total operation time were significantly reduced compared to our control group (Fig. 4). Time savings varied from 14 (cross-clamping) up to 20 min (operation). Usually 15 to 20 sutures are used for fixation of an annuloplasty ring in our clinic. The time saving in our study is consistent with the results obtained by Lee et al. [10], in which the Cor- Knot device was compared with hand-tied knots using a knot pusher in an ex vivo porcine minimally invasive simulation model. A significant reduction in time of almost one minute was obtained for the Cor-Knot device for one knot (12.4 vs 71.1 seconds per knot, *p* = 0.001) as compared with the manually tied knot using the traditional knot pusher. Another important feature of the Cor-Knot device is the release of the fastener, which is only possible under tension, thereby guaranteeing the requested tissue attachment. Undesirable loose knots, which can occur by placing hand-tied knots with a knot pusher with additional time loss, are eliminated.

Saving time during cross-clamping provides the surgeon with more flexibility in case of a suboptimal mitral repair result and the need for a second or even a third run of cross-clamping with additional readjustment procedures. Increased cross-clamp and CPB time have been shown to be independent predictors of morbidity and mortality in cardiac surgery [11, 12].

In our study, no differences in clinical outcome, i.e. minor or major adverse events, were noted. All patients safely left our hospital for cardiac rehabilitation programs. All underwent repair of the mitral valve in the first run (one cross-clamping period) with the anticipated good operative results and therefore no significant differences in outcome are to be expected, especially given the lack of power with only 60 included patients.

Another important issue is economics: The Cor-Knot device with the application of 20 fasteners costs around 1000 Swiss Francs, which has to be compared with the operative time saving of 15 – 20 min. In Basel, one minute in the cardiac surgery OR costs around 80 Swiss Francs, which means a reduction in OR costs of between 1200 and 1600 Swiss Francs and a profit of 200–600 Swiss Francs.

## Conclusion

Our short-term results indicate a safe, reliable and fast application of the novel automated fastener device in combination with significant time savings in cardioplegic arrest and cardiopulmonary bypass.
